# Cellular Stress Responses in Cancer and Cancer Therapy

**DOI:** 10.3389/fonc.2014.00304

**Published:** 2014-10-29

**Authors:** Megan Chircop, Daniel Speidel

**Affiliations:** ^1^Children’s Medical Research Institute, Westmead, NSW, Australia; ^2^Sydney Medical School, University of Sydney, Sydney, NSW, Australia

**Keywords:** apoptosis, DNA damage, DNA repair, chemotherapy, cellular stress, tumorigenesis, therapeutic targets, therapy resistance

In response to stress, cells activate so-called checkpoints – complex signaling pathways that induce a plethora of cellular outcomes. Checkpoints primarily initiate cell cycle arrest to provide the cell with time to repair the damage. However, if the damage is too severe then cells can permanently arrest the cell cycle (senescence) or trigger cell death, thereby preventing the transmission of genetic defects. These responses are pivotal for tumor suppression as all of these outcomes result in restriction of the growth and/or elimination of damaged and pre-malignant cells. Thus, a large number of anti-cancer agents target specific components of stress response signaling pathways with the aim of causing tumor regression by stimulating cell death or at least stopping cell growth. However, the efficacy of these agents is often impaired by mutations in genes that are involved in stress-responsive signaling pathways. Moreover, these cancer-specific genetic defects often contribute to resistance against chemotherapeutic agents and/or radiotherapy. Modulating the outcome of cellular stress responses toward cell death in tumor cells without affecting surrounding normal cells is thus one of the ultimate aims in the development of new cancer therapeutics. To achieve this aim, a detailed understanding of cellular stress response pathways and their aberrations in cancer is required.

The Research Topic titled “Molecular mechanisms of cellular stress responses in cancer and their therapeutic implications” features 11 articles that reflect the broadness and complexity of the processes induced by cellular stress. It begins with reviews on four different proteins/protein families that are critical for cellular stress responses and as such are important for both cancer development and the response to cytotoxic therapies.

Knippschild and colleagues discuss the complex functions of the casein kinase 1 (CK1) family and describe in depth how members of this family regulate signaling cascades that are relevant for the pathogenesis of inflammatory and proliferative diseases and, beyond this, for neurodegenerative disorders as well. They also summarize current knowledge on therapeutic modulation of CK1 activity and existing inhibitors (1).

In addition to phosphorylation, other post-translational modifications are an effective means to modify the activity of cellular proteins and hence respond to stress signals. Polonio-Valon et al. focus on the peptidyl-prolyl *cis/trans* isomerase Pin1, an enzyme that can induce conformational changes in its substrate proteins. In their article, they highlight the interactions of Pin1 with key proteins relevant to cancer and cancer therapy and discuss how Pin1 specifies cell fate decision in response to DNA damage (2).

One of the key proteins in cellular stress signaling is the tumor suppressor p53 and any collection of articles dealing with stress responses would be incomplete without an article on p53. Mueller and colleagues give a brief overview on the extensive literature on p53 and its family members, p63 and p73 with a specific focus on therapeutic implications (3).

Parker et al. discuss another well known protein family, the tubulins and their interacting partners. Tubulins are the building blocks of microtubules and therefore responsible for cell movement, intracellular trafficking, and cell division. They are also the target of a specific class of chemotherapeutics. As this article points out, microtubules and associated proteins play an important role in a range of cellular stress responses (4).

Understanding cellular processes that are differentially regulated in cancerous versus normal cells is a prerequisite for exploiting them therapeutically. Three articles focus on this aspect.

A hallmark of cancer as a proliferative disorder is the increased number of cell divisions and a high mitotic index. Mitotic cells respond differently to stress signals than interphase cells due to their condensed chromosomes. Burgess and colleagues review the pathways and outcomes activated by mitotic cells in response to stress and describe how this influences efficacy of chemotherapeutic drugs, especially those in the anti-mitotic class (5).

Abnormal DNA content is another common hallmark of cancer cells that has been recognized for a long time. In their hypothesis and theory article, Coward and Harding summarize evidence that links the acquisition of multiple chromosome copies (polyploidy) to tumor evolution and chemotherapy resistance. They argue that these polyploid cells themselves are critical drug targets (6).

Double-strand breaks are also prevalent in many cancer cells due to their increased proliferation and impaired DNA repair programs. Jekimovs et al. review the two DNA repair pathways activated by DNA double-strand-breaks and discuss the successes and failures of pre-clinical and clinical trials aiming to modulate these pathways (7).

Finally, four articles highlight some of the many factors that influence the success of cancer therapy with cytotoxic agents.

One of the most challenging problems is tumor heterogeneity, a topic discussed by Renovanz and Kim who argue that there is much to learn to be able to treat cancer patients more effectively (8).

Tumor hypoxia is another problematic aspect in many solid tumors as this has been linked to resistance against radiation and chemotherapy. In their original research article, Ontikatze and colleagues characterize a specific drug, dihydroartemisinin, that may be instrumental in overcoming therapy resistance of hypoxic tumors (9).

Hormones can also affect the response to cytotoxic agents and this seems particularly obvious for estrogen. Caldon describes the complicated relationship of estrogen and DNA damage signaling in breast cancer and proposes that estrogen receptor signaling suppresses effective DNA repair and apoptosis in favor of proliferation (10).

Breast cancer is also the focus of the original article by Quante and colleagues who report new insights into the process leading to hyperplastic lesions in the mammary gland obtained from the analysis of a transgenic mouse model (11).

This collection of articles highlights some of the advances made in understanding the molecular mechanisms of cellular stress responses and the implications of this for cancer biology. Research in this field has already enabled improved clinical outcomes for cancer patients and we are hopeful that with continued investigation of this topic more discoveries will be translated into even better cancer treatments.

## Conflict of Interest Statement

The authors declare that the research was conducted in the absence of any commercial or financial relationships that could be construed as a potential conflict of interest.
